# Metabolic correlates of reserve and resilience in MCI due to Alzheimer's Disease (AD)

**DOI:** 10.1186/s13195-018-0366-y

**Published:** 2018-04-03

**Authors:** Matteo Bauckneht, Andrea Chincarini, Roberta Piva, Dario Arnaldi, Nicola Girtler, Federico Massa, Matteo Pardini, Matteo Grazzini, Hulya Efeturk, Marco Pagani, Gianmario Sambuceti, Flavio Nobili, Silvia Morbelli

**Affiliations:** 1 Department of Health Sciences (DISSAL), University of Genoa, Genoa, Italy; 2 Nuclear Medicine Unit, Polyclinic San Martino Hospital, Genoa, Italy; 3 National Institute of Nuclear Physics (INFN), Genoa, Italy; 4 Department of Neuroscience (Dipartimento di Neuroscienze, Riabilitazione, Oftalmologia, Genetica e Scienze Materno-Infantili [DINOGMI]), University of Genoa, Genoa, Italy; 5 Neurology Clinics, San Martino Hospital Polyclinic, Genoa, Italy; 6 Institute of Cognitive Sciences and Technologies (ICST), Consiglio Nazionale delle Ricerche (CNR), Rome, Italy; 7 Department of Nuclear Medicine, Karolinska Hospital Stockholm, Stockholm, Sweden

**Keywords:** ^18^F-FDG PET, Alzheimer’s disease, Mild cognitive impairment, Cognitive Reserve, Resilience

## Abstract

**Background:**

We explored the presence of both reserve and resilience in late-converter mild cognitive impairment due to Alzheimer’s disease (MCI-AD) and in patients with slowly progressing amyloid-positive MCI by assessing the topography and extent of neurodegeneration with respect to both “aggressive” and typically progressing phenotypes and in the whole group of patients with MCI, grounding the stratification on education level.

**Methods:**

We analyzed 94 patients with MCI-AD followed until conversion to dementia and 39 patients with MCI who had brain amyloidosis (AMY+ MCI), all with available baseline ^18^F-fluorodeoxyglucose positron emission tomography (FDG-PET) results. Using a data-driven approach based on conversion time, patients with MCI-AD were divided into typical AD and late-converter subgroups. Similarly, on the basis of annual rate of Mini Mental State Examination score reduction, AMY+ MCI group was divided, obtaining smoldering (first tertile) and aggressive (third tertile) subgroups. Finally, we divided the whole group (MCI-AD and AMY+ MCI) according to years of schooling, obtaining four subgroups: poorly educated (Low-EDUC; first quartile), patients with average education (Average-EDUC; second quartile), highly educated (High-EDUC; third quartile), and exceptionally educated (Except-EDUC; fourth quartile). FDG-PET of typical AD, late converters, and aggressive and smoldering AMY+ MCI subgroups, as well as education level-based subgroups, were compared with healthy volunteer control subjects (CTR) and within each group using a two-samples *t* test design (SPM8; *p* < 0.05 family-wise error-corrected).

**Results:**

Late converters were characterized by relatively preserved metabolism in the right middle temporal gyrus (Brodmann area [BA] 21) and in the left orbitofrontal cortex (BA 47) with respect to typical AD. When compared with CTR, the High-EDUC subgroup demonstrated a more extended bilateral hypometabolism in the posterior parietal cortex, posterior cingulate cortex, and precuneus than the Low- and Average-EDUC subgroups expressing the same level of cognitive impairment. The Except-EDUC subgroup showed a cluster of significant hypometabolism including only the left posterior parietal cortex (larger than the Low- and Average-EDUC subgroups but not further extended with respect to the High-EDUC subgroup).

**Conclusions:**

Middle and inferior temporal gyri may represent sites of resilience rather than a hallmark of a more aggressive pattern (when hypometabolic). These findings thus support the existence of a relatively homogeneous AD progression pattern of hypometabolism despite AD heterogeneity and interference of cognitive reserve. In fact, cortical regions whose “metabolic resistance” was associated with slower clinical progression had different localization with respect to the regions affected by education-related reserve.

**Electronic supplementary material:**

The online version of this article (10.1186/s13195-018-0366-y) contains supplementary material, which is available to authorized users.

## Background

^18^F-fluorodeoxyglucose positron emission tomography (FDG-PET) and structural magnetic resonance imaging (MRI) have been demonstrated to reflect cognitive function and are considered progression biomarkers in patients with Alzheimer’s disease (AD) [1]. Moreover, given their capability to demonstrate neurodegeneration in vivo, both FDG-PET and MRI have significantly contributed to the understanding of cognitive reserve-related adaptive mechanisms [2–4]. In fact, given a particular level of imaging-assessed brain damage, cognitive reserve could hypothetically be defined as the difference between an individual’s expected and actual cognitive performance [5]. However, the concept of cognitive reserve and the capability of FDG-PET and MRI to capture reserve mechanisms are somehow in contrast to the emerging role and value of these techniques as predictors of clinical disease milestones, such as time to conversion from the mild cognitive impairment (MCI) to the dementia stage. Moreover, whereas a large body of literature has been devoted to assessment of the value of FDG-PET in the prediction of further cognitive decline in MCI for diagnostic purposes, only the identification and localization of regions whose metabolism is able to predict the speed of progression in patients with mild cognitive impairment due to Alzheimer’s disease (MCI-AD) may allow researchers to further address the existence of a specific interference due to cognitive reserve [6–9]. We recently demonstrated the role of FDG-PET as a significant progression biomarker in a naturalistic group of patients with MCI-AD by demonstrating that baseline middle and inferior temporal metabolism is able to capture the speed of conversion to AD dementia regardless of confounding factors such as age and education [10]. However, in our previous analysis, we did not further explore whether metabolic levels in these regions represent a marker of more aggressive disease (i.e., more marked hypometabolism accelerating conversion) or a potential site of resilience (i.e., relatively preserved metabolic levels corresponding to resistance to neurodegeneration delaying conversion in MCI-AD). In fact, whereas in patients with AD cognitive reserve is supposed to protect against the cognitive consequences of AD pathology and not against the accumulation of the pathology itself, resilience may refer to both reserve and maintenance mechanisms (i.e., resistance to brain neurodegeneration despite the presence of AD pathology) [11–13]. Although several lines of evidence support the idea that despite a greater amount of neurodegeneration, the clinical phenotype of AD in highly educated individuals may be similar to that found in patients with lower education and less pathology [14], the existence of an influence of protective factors and reserve proxies on the aggregation of AD pathology and consequent neurodegeneration is an ongoing research issue (*see* [15, 16] for detailed reviews). Accordingly, the existence of maintenance mechanisms in late-converter patients with MCI-AD would represent a further source of complexity in the construct of brain reserve and might explain the lack of influence of mere statistical adjustments (such as covarying for the years of education) on the value of baseline brain metabolism as a predictor of disease progression.

In this study, we thus aimed to explore the presence of both reserve and resilience in late-converter patients with MCI-AD and in patients with slowly progressing amyloid-positive MCI by assessing (1) topography and extent of neurodegeneration with respect to both “aggressive” and typically progressing phenotypes and (2) topography and extent of neurodegeneration in the whole group of patients with MCI, grounding the stratification on education level. In particular, because a larger group of patients with MCI-AD (with respect to our previous study [10]) and a new independent group of patients with amyloid-positive MCI were included in the present study, all the analyses were performed with a voxel-based whole-brain approach to independently confirm the location of cortical regions affecting clinical progression and to assess the topography of regions whose metabolism is more strictly influenced by reserve-related mechanisms.

## Methods

### Selection of participants and clinical neuropsychological assessment

This study was approved by the institutional review board of our memory clinic, and all subjects signed an informed consent form. Study participants were recruited from two different cohorts, both derived from the naturalistic population of our memory clinic. The selection procedure for the first group (group A) was intended to retrospectively identify a consecutive series of patients with the following characteristics: (1) evaluated for the first time at our memory clinic in the frame of MCI suspected to be AD-related, (2) underwent brain FDG-PET at baseline during the first diagnostic workup, and (3) followed at least until clinical conversion to AD dementia with regular control visits allowing the definition of conversion time with a degree of uncertainty shorter than 6 months. The presence of dementia was established by clinical interview with the patient and informants, using questionnaires for activities of daily living, instrumental activities of daily living, and the Clinical Dementia Rating (CDR). Only patients with dementia attributed to AD according to the National Institute on Aging-Alzheimer’s Association criteria were included [17]. We thus aimed to investigate this group to clarify the interactions between baseline imaging and clinical features that influenced rate of conversion from MCI to AD dementia stage. Accordingly, we queried our local database from its original composition in 2007 to include patients converted at different follow-up times after baseline evaluation. Because the clinical use of amyloid positron emission tomography (AMY-PET) was introduced in Italy in 2014, a large majority of patients evaluated for the first time between 2007 and 2014 did not undergo AMY-PET. To avoid heterogeneity, the selection of patients for this group was limited to patients recruited between 2007 and 2014 (group A, MCI clinically converted to AD, or MCI-AD).

The second group of patients (group B) was selected to perform the same analyses as in group A in an independent group of patients with MCI who had a positive result for a brain amyloidosis biomarker (AMY+ MCI). The selection procedure was intended to retrospectively identify a consecutive series of patients with MCI-AD with the following characteristics: (1) underwent FDG-PET close to the baseline examination in our center at the stage of MCI, (2) had amyloid positivity (AMY+ MCI) confirmed in vivo, and (3) had more than two time points for Mini Mental State Examination (MMSE) evaluation and clinical neuropsychological follow-up of at least 1 year after baseline FDG-PET. The speed of clinical progression was thus used as a criterion to classify patients as having “aggressive” or “smoldering” AMY+ MCI (*see below*).

For both groups, we selected patients with single- or multidomain amnestic MCI. Accordingly, to be included, patients had to demonstrate impairment on a memory test, either with (multidomain amnestic MCI) or without (single-domain amnestic MCI) impairment in other cognitive domains but not demented, thus corresponding to Petersen and Negash’s MCI criteria [18]. It has to be underlined that, especially given that our memory clinic is a tertiary center, some of the patients had previously been evaluated in other centers and classified as having subjective impairment or MCI before arrival at our memory clinic. This information has been noted in our medical records, and the time lapse between earlier evaluations in other centers and our “baseline” evaluation ranged from 6 months to 2 years. However, the reliability of the information related to the time elapsed between the onset of memory complaints and our baseline evaluation was largely variable across patients (i.e., simply reported by the caregiver or available through historical clinical documentation). Overall clinical experience tells us that it is a poorly reliable measure because the time of symptom onset largely varies even in the same patient, depending on whom the patient is interviewed by. Typically, the patient is poorly reliable, and the patient’s relatives report discrepant stories, which in turn differ from the patient’s. For these reasons, we did not include this variable in the present analyses.

Patients underwent a neuropsychological test battery at baseline, including tests for language, visuoconstruction, attention, cognitive flexibility, verbal episodic memory, spatial memory, and working memory. Global cognition was assessed using the MMSE in all patients. The test battery was tailored according to the clinical presentation and could differ among patients also in consideration of the large time span over which the batteries were administered. Patients evaluated for the first time between 2007 and 2014 underwent a battery of neuropsychological tests that varied with the course of time and according to the clinical presentation and suspicion [20]. These tests mainly include: (1) categorical and phonological verbal fluency for language with the Token Test when there was a suspicion of comprehension deficit; (2) Trail Making Test A and B and the Stroop Color and Word Test or the Wisconsin Card Sorting Test for executive function; (3) figure copying of the Mental Deterioration Battery (simple copy and copy with guiding landmarks) or copy and delayed recall of the Rey figure and the Clock Completion Test to assess visuospatial ability; (4) Rey Auditory Verbal Memory Test (immediate and delayed recall) or the Grober-Buschke Free and Cued Selective Reminding Test, or the Babcock Story Recall Test for verbal memory, and Corsi block-tapping test to investigate spatial memory; and (5) digit span (forward) and digit symbol or visual search in attention matrices to assess attention and working memory. The neuropsychological test scores were corrected for age and education according to published normative data in the Italian language. A z-score less than − 1.5 (or the equivalent score of 0 for those tests without a normal distribution [19]), computed on the Italian normative values of each test and corrected for age and education, was established for impairment in a specific cognitive domain.

Exclusion criteria included previous or current major psychiatric disorder and neurological disease; severe and uncontrolled arterial hypertension; diabetes mellitus; renal, hepatic, or respiratory failure; anemia; and malignancy. A depressive trait was not an exclusion criterion, but a 15-item Geriatric Depression Scale score ≤ 10 was required for inclusion. Patients with MRI evidence of major stroke or brain mass were excluded, with white matter hyperintensities, leukoaraiosis, and lacunae not constituting exclusion criteria if the Wahlund score was < 3 in all regions [21]. The modified Hachinski ischemic score [22] < 3 in all patients. Patients fitting the criteria for vascular cognitive impairment [23] were excluded.

### Control subjects

The control subjects were 48 healthy volunteers (CTR) who gave their informed consent to participate. Their healthy condition was carefully checked by means of general medical history, clinical examination, and the same exclusion criteria used for the study groups, with the exception of cognitive complaints. The MMSE was administered, and only subjects with a normal score (i.e., > 26) were considered. Moreover, only subjects with a CDR of 0 were included. These subjects underwent the same neuropsychological battery as the study groups, as well as FDG-PET and MRI. The control subjects were chosen from the same age range and had a gender distribution and education level similar to those of the study groups. The main characteristics of the CTR group are listed in Table 1.

**Table 1:** Demographic and main clinical characteristics of the study groups

Characteristics	CTR (*n* = 48)	MCI-AD (*n* = 94)	AMY+ MCI (*n* = 39)
Age, years	69.0 ± 9.7	75.3 ± 5.7	74.2 ± 6.4
Sex			
Male	12	42	21
Female	36	52	18
Education, years	10.0 ± 4.0	10.1 ± 2.0	10.5 ± 4.1
ApoE ε4 allele status^a^	N/A	N/A	14/22^b^
Baseline NPS			
MMSE	29.0 ± 0.9	26.0 ± 1.1	27.1 ± 2.6
Follow-up duration, years	–	3.2 ± 1.5	2.3 ± 1.0
Conversion time, months	–	23.2 ± 16.2 (range 6–98)	–
Follow-up MMSE score	–	22.3 ± 2.0	25.9 ± 3.4
ΔMMSE/year		− 1.5 ± 1.2	− 0.6 ± 0.4

*Abbreviations: AMY+ MCI* Patients with mild cognitive impairment who had a positive result for a brain amyloidosis biomarker, *ApoE* Apolipoprotein E, *CTR* Healthy volunteer control subjects, *MCI-AD* Patients with mild cognitive impairment due to Alzheimer’s disease, *MMSE* Mini Mental State Examination, *ΔMMSE* Reduction in Mini Mental State Examination score, *NPS* Neuropsychology

Data are presented as mean ± SD

^a^Either as homozygous or heterozygous for ApoE ε4 allele

^b^Available in 22 of 39 patients (3 of 22 homozygous and 11 of 22 heterozygous for ApoE ε4 allele) to date

### Patient grouping

#### Group A

Ninety-four consecutive subjects with MCI matched the study criteria and were included in group A (age 75.3 ± 5.7 years; 52 females, 42 males; baseline MMSE score 26.0 ± 1.1). They converted to AD dementia 6 to 98 months after their baseline visit (mean 23.2 ± 16.2) (*see* Table 1 for further clinical details). We aimed to characterize the time profile of their conversion to AD dementia. In this respect, to identify the progressing phenotype in the short and medium term (“typical AD”) and in late-converter patients, we divided the MCI-AD group by implementing a data-driven approach based on their conversion time. Conversion time alone was used as a clustering variable, and hierarchical clustering was applied with an unweighted average distance linkage method.

#### Group B

Thirty-nine consecutive subjects with AMY+ MCI matched the study criteria (age 74.2 ± 6.4 years; 21 females, 18 males; baseline MMSE score 27.1 ± 2.6; mean follow-up 2.3 ± 1 years, range 1–3 years) (*see* Table 1 for further clinical details). As per the inclusion criteria, all patients were AMY+ according to AMY-PET or cerebrospinal fluid (CSF) analysis. Specifically, the information on brain amyloidosis was obtained by AMY-PET in 30 patients and by CSF assay in 9 patients. We chose to use the MMSE score as a marker of global cognition. The MMSE was administered with all patients because the patients in a time span of almost 10 years did not always undergo the same neuropsychological tests.

Patients’ annual rate of MMSE reduction (ΔMMSE) was computed by taking into consideration the baseline score and the fact that each patient had several neuropsychological examinations, ranging from a minimum of three to a maximum of five. The availability of more than two time points allowed us to better estimate ΔMMSE. We computed ΔMMSE by linear regression, which allowed us to assess the ΔMMSE CI, too. We then used a linear model with ΔMMSE as the dependent variable and the baseline MMSE, patient sex, and patient age as covariates. In order to make use of the availability of several time points, we used weights on the baseline MMSE that were inversely proportional to the CI of the ΔMMSE. The only significant covariate was the baseline MMSE (*p* < 0.0001), which was then used to compute a corrected ΔMMSE. Then, to highlight the progressing phenotypes and thus to identify smoldering and aggressive AMY+ MCI according to the annual rate of ΔMMSE alone, a data-driven approach based on patients’ annual rate of ΔMMSE was implemented as previously done for group A and for conversion time.

#### Education-based subgroups

To explore the interplay between education and conversion time, we took into account the entire group of patients with MCI (group A + group B). Because we specifically aimed to address the existence of education-related reserve mechanisms in the same group of patients with MCI the clinical aggressiveness of which was previously evaluated, we tried to stratify and isolate as much as possible patients with MCI who were highly educated. For this reason, we independently divided all patients into quartiles according to their years of formal education, thus obtaining groups that were poorly educated (first quartile; Low-EDUC), of average education level (second quartile; Average-EDUC), were highly educated (High-EDUC; third quartile), or were exceptionally educated (fourth quartile; Except-EDUC group).

### Amyloidosis and neurodegeneration biomarker assessment

#### FDG-PET protocol

FDG-PET data were acquired according to the guidelines of the European Association of Nuclear Medicine as detailed in Additional file 1 [24].

#### Brain amyloidosis assessment

Given the naturalistic nature of the groups, assessment of brain amyloidosis was previously obtained according to patients’ preferences and PET tracer availability. AMY-PET imaging acquisition and visual reading were performed according to published guidelines [25] and manufacturers’ instructions as detailed in Additional file 1. Standardized uptake value ratios (SUVRs) using the whole cerebellum as the reference region were calculated, and scan positivity was established, using published cutoffs for each tracer [26, 27] (*see* reference [28] and Additional file 1 for further details). To provide a further SUVR-independent method to define AMY-PET positivity, the scans were also evaluated by means of an original semiquantitative tool developed and validated in our laboratory [28]. Briefly, this tool, named *ELBA*, involves minimal image preprocessing and does not rely on small, specific regions of interest (ROIs). It evaluates the whole brain and delivers a geometric/intensity score to be used for both ranking and dichotomous assessment [28]. ELBA scoring has been demonstrated to significantly correlate with SUVR values, and the longitudinal analysis estimated a test-retest error of ~ 2.3% on the Alzheimer's Disease Neuroimaging Initiative database (*see* [28] for further details). All the patients with MCI included in group B were positive on both visual and semiquantitative assessment with both SUVR and ELBA.

#### CSF assay

Single-parameter colorimetric enzyme-linked immunosorbent assay (ELISA) kits (Innogenetics, Ghent, Belgium) were used to measure amyloid-β 42 (Aβ_42_). ELISA assay of CSF was performed according the current standard [29] using the INNOTEST® (Fujirebio, Ghent, Belgium) commercial kit. The status of AMY+ was defined with a value below the cutoff in our center (< 600 pg/ml). Levels of protein tau and a phosphorylated form of tau at residue 181 were also measured. Further details can be found in Additional file 1.

### Image processing and statistical analysis

Preprocessing of FDG-PET images was performed using the default routine of a Statistical Parametric Mapping (SMP) stand-alone version (SPM8; Wellcome Centre for Human Neuroimaging, UCL, London, UK) [30]. However, the H_2_^15^O SPM default template was replaced with a dementia-optimized brain FDG-PET template as described by Della Rosa and colleagues [31]. The spatially normalized set of images was then smoothed with an 8-mm isotropic Gaussian filter. Characterization of the degree and topography of neurodegeneration in late converters of group A and in those with smoldering AMY+ MCI in group B was obtained by means of two-samples *t* test of SPM8 running the following analyses:

#### Group A

1. CTR versus subgroups of patients divided according their conversion time, thus including the late-converter patients and patients with “typical AD”
2. Late-converter subgroup versus patients with “typical AD”

#### Group B

1. CTR versus subgroups of patients divided according to the annual speed of reduction of MMSE score
2. Smoldering versus aggressive AMY+ MCI

In all the analyses, we included age, sex, and education as nuisance variables.

### Education-based analyses

To evaluate the relationship between education on one side and both severity and topography of neurodegeneration on the other, a two-samples *t* test design was used to compare the Low-EDUC (*n* = 33; mean years of education 6.1 ± 1.0), Average-EDUC (*n* = 33; mean years of education 8.9 ± 1.4), High-EDUC (*n* = 33; mean years of education 12.4 ± 2.3), and Except-EDUC (*n* = 34; mean years of education 17.4 ± 1.0) subgroups with CTR. Age, sex, and MMSE score were used as nuisance variables. Owing to the unavoidable patient discretization, partitions did not contain the exact same number of patients. *See* Table 2 for further details on education-based subgroups.

**Table 2:** Demographic and main clinical characteristics of education-based groups

Characteristics^a^	Low-EDUC (*n* = 33)	Average-EDUC (*n* = 33)	High-EDUC (*n* = 33)	Except-EDUC (*n* = 34)
Age, years	74.1 ± 4.9	75.4 ± 7.2	73.6 ± 3.2	71.3 ± 10
Sex				
Male	13	8	19	23
Female	20	25	14	11
Education, years	6.1 ± 1.0	8.9 ± 1.4	12.4 ± 2.3	17.4 ± 1.0
Baseline NPS				
MMSE	26.7 ± 3.9	26.0 ± 2.6	27.2 ± 2.3	27.3 ± 1.9
ΔMMSE score/year	− 1.2 ± 1.3	− 1.6 ± 1.5	− 0.9 ± 0.8	− 1 ± 0.8

*Abbreviations: MMSE* Mini Mental State Examination, *ΔMMSE* Mini Mental State Examination reduction, *NPS* Neuropsychology

Data are presented as mean ± SD

^a^The whole group (*n* = 133) was divided into quartiles: Low-EDUC, first quartile; Average-EDUC, second quartile; High-EDUC, third quartile; and Except-EDUC, fourth quartile

For all SPM analyses, the significance threshold was set at *p* < 0.05 with family-Wise error correction at both peak and at cluster levels. Only significant clusters containing at least 100 voxels were taken into consideration. For both analyses, coordinates of significant clusters in Montreal Neurological Institute (MNI) space were converted into Talairach coordinates, and corresponding gray matter regions and Brodmann areas (BAs) were identified. Correction of MNI coordinates to match the Talairach coordinates was achieved by using BrainMap GingerALE 2.3 [32]. BAs were then identified at a range of 0 to 3 mm from the corrected Talairach coordinates of the SPM output isocenters after importing the corrected coordinates by means of the Talairach client (http://www.talairach.org/index.html).

## Results

### Patient groups

#### Group A

**Fig. 1:**
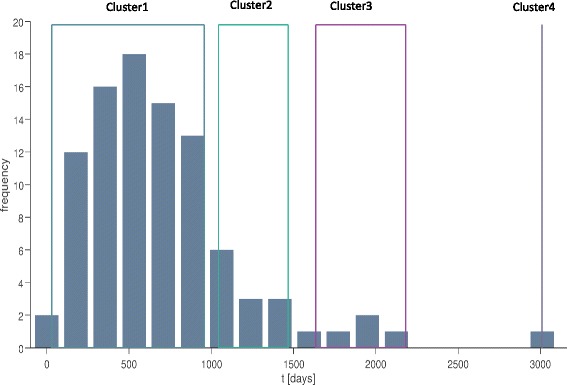
Clusters of conversion time in patients with mild cognitive impairment who converted to Alzheimer’s disease during follow-up (group A). Calinski-Harabasz and Silhouette evaluations suggested an optimal number of three clusters plus an outlier (indicated as cluster 4). Conversion time cutoff estimations are (in days): 1000 (between clusters 1 and 2) and 1550 (between clusters 2 and 3). The majority of patients (*n* = 76) converted to dementia within 27 months and were included in the first cluster. Given the numerical predominance of this conversion time-based group with respect to the others, we labeled the group as “typical Alzheimer’s disease,” whereas we considered patients belonging to clusters 2, 3, and 4 as late-converter patients

Both the Calinski-Harabasz and Silhouette evaluations suggested an optimal number of three clusters plus an outlier (indicated as cluster 4 in Fig. 1). Conversion time cutoff estimations are (in days): 1000 (between clusters 1 and 2) and 1550 (between clusters 2 and 3) (*see* Fig. 1). The majority of patients (*n* = 76) converted to dementia within 27 months and were included in the first cluster. Given the numerical predominance of this conversion time-based group with respect to the other ones, we labeled the group as “typical AD” (age 75.2 ± 6.7 years; 36 females; 10.2 ± 4.5 years of education; baseline MMSE score 27.5 ± 1.9), and we considered patients belonging to clusters 2, 3, and 4 as late-converter patients (*n* = 18; age 75.7 ± 6.3 years; 16 females; 9.9 ± 4.7 years of education; baseline MMSE score 26.5 ± 1.1).

#### Group B

The mean annual rate of ΔMMSE for the whole AMY+ MCI group was 1.26 ± 1.76 (*see* Table 1 for clinical details). In this case, both the Calinski-Harabasz and Silhouette evaluations suggested an optimal number of three clusters (*see* Additional file 2: Figure S1); however, given the short follow-up available for group B, this kind of analysis was more suitable for identifying the aggressive subgroup (ΔMMSE > 4.5 points/year). In fact, the majority of patients showed a ΔMMSE between 0 and 1 point/year. Because we were specifically interested in the characterization of smoldering patients (by analogy to late-converter patients in group A), we considered that although more arbitrary, a division into tertiles based on their annual rate of ΔMMSE would have been more suitable for our aims. For this reason, patients were then divided into tertiles. AMY+ MCI patients belonging to the first tertiles were considered as “smoldering MCI” (*n* = 13). Notably, after correction for age and baseline MMSE, the annual rate of MMSE change in all the patients belonging to this tertile showed no reduction, and actually a small but measurable increase in MMSE score was highlighted (mean annual rate of MMSE change + 0.7 ± 0.5; range 0.2–1.8). By contrast, patients belonging to the third tertiles were considered as “aggressive MCI” tertiles (*n* = 13; mean annual rate of ΔMMSE − 2.2 ± 1.8; range − 0.6 to − 1.8). Patients belonging to the second tertile had a mean annual rate of ΔMMSE of − 0.2 ± 0.2 (range + 0.1 to − 0.5; *n* = 13; age 75.3 ± 5.8 years; 7 females; 9.5 ± 2.9 years of education; baseline MMSE score 27.6 ± 1.7). *See* Table 3 for further details on the aggressive and smoldering AMY+ MCI groups.

**Table 3:** Demographic and main clinical characteristics of “typical Alzheimer’s disease,” late converters, and “smoldering” and “aggressive” mild cognitive impairment

Characteristics	Typical AD (*n* = 76)	Late converters (*n* = 18)	Smoldering MCI (*n* = 13)	Aggressive MCI (*n* = 13)
Age, years	75.2 ± 6.7	75.7 ± 6.3	74.8 ± 4.8	73.8 ± 8.0
Sex				
Male	12	2	8	7
Female	36	16	5	6
Education, years	10.2 ± 4.5	9.9 ± 4.7	11.3 ± 5.3	10.7 ± 3.9
Baseline MMSE	27.5 ± 1.9	26.5 ± 1.1	27.2 ± 3.1	26.6 ± 3.0
ΔMMSE score/year	− 1.6 ± 1.5	− 1.1 ± 0.9	+ 0.7 ± 0.5	− 2.2 ± 1.8

*Abbreviations: AD* Alzheimer’s disease, *MCI* Mild cognitive impairment, *MMSE* Mini Mental State Examination, *ΔMMSE* Mini Mental State Examination reduction

### Topography and extent of neurodegeneration and resilience in relation to speed of progression

#### Group A

**Fig. 2:**
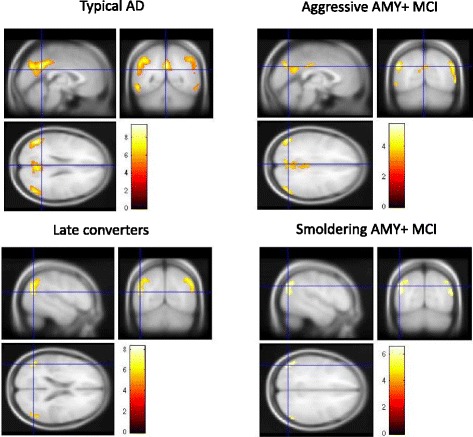
Cortical hypometabolism in patients with mild cognitive impairment who converted to Alzheimer’s disease dementia during follow-up (MCI-AD) and patients with mild cognitive impairment with brain amyloidosis (AMY+ MCI) with respect to control subjects. The typical Alzheimer’s disease (AD) group showed a wide area of hypometabolism involving the posterior parietal cortex and the precuneus in both hemispheres as well as the middle and superior occipital gyri, and also involving the posterior cingulate cortex in the left hemisphere (Brodmann areas [BAs] 7, 19, 30, 31, and 40 BA 22), whereas hypometabolism in late converters was limited to the bilateral posterior parietal cortex (BAs 7 and 40). Similarly, in the AMY+ MCI group, those with aggressive AMY+ MCI were characterized by a bilateral extended area of hypometabolism in the left superior temporal gyrus and posterior cingulate cortex, as well as in the posterior parietal cortex, lateral cuneus, and precuneus in both hemispheres (BAs 7, 18, 19, 22, 31, and 40), whereas patients with smoldering mild cognitive impairment (MCI) were characterized by hypometabolism limited to smaller clusters in the bilateral posterior parietal cortex (BA 40). *See* Table 4 and Additional file 3: Table S1 for details of coordinates and z-scores. Clusters with significant hypometabolism are shown superimposed on a multiple subject averaged magnetic resonance imaging template. The color bars indicate the level of *z*-scores for significant voxels

As expected, the typical AD group was characterized by a large bilateral area of hypometabolism involving the posterior parietal cortex and precuneus in both hemispheres, as well as the middle and superior occipital gyri and the posterior cingulate cortex in the left hemisphere (BAs 7, 19, 30, 31, and 40 BA 22). By contrast, compared with CTR, brain metabolism in late converters was less extended and limited to the bilateral posterior parietal cortex (BAs 7 and 40). *See* Fig. 2 and Table 4 for further details.

**Table 4:** Whole-brain voxel-based analyses of ^18^F-fluorodeoxyglucose positron emission tomographic images in SPM8

	Cluster level		Peak level					
Cluster extent	Corrected *p* value	Cortical region	Maximum z-score		Talairach coordinates		Cortical region	BA
Comparison between CTR and patients with “typical” AD								
15,247	0.0001	L-parietal	8.01	− 8	− 73	55	Precuneus	7
		L-limbic	6.05	− 10	− 43	39	Posterior cingulate gyrus	31
		L-parietal	5.04	− 42	− 74	44	Precuneus	19
		L-parietal	5.98	− 44	− 68	49	Inferior parietal lobule	7
		R-parietal	4.89	50	− 47	28	Inferior parietal lobule	40
		L-parietal	4.83	− 12	− 47	34	Precuneus	31
		R-occipital	4.64	30	− 75	24	Precuneus	31
		R-parietal	4.19	10	− 64	47	Precuneus	7
		L-parietal	4.08	− 61	− 53	34	Supramarginal gyrus	40
		L-temporal	4.08	− 65	− 44	10	Superior temporal gyrus	22
		L-parietal	3.93	− 57	− 38	50	Inferior parietal lobule	40
		L-limbic	3.53	− 18	− 58	16	Posterior cingulate gyrus	30
		L-parietal	3.43	− 57	− 38	50	Inferior parietal lobule	40
Comparison between CTR and late-converter patients								
983	0.006	L-parietal	8.83	− 57	− 38	50	Inferior parietal lobule	40
		R-parietal	6.09	50	− 47	28	Inferior parietal lobule	40
		L-parietal	4.01	− 44	− 68	49	Inferior parietal lobule	7

*Abbreviations: AD* Patients with mild cognitive impairment who clinically converted to dementia of Alzheimer’s type during follow-up, *BA* Brodmann area, *CTR* Healthy volunteer control subjects

*p* < 0.05 corrected for multiple comparisons with the family-wise error correction at both peak and cluster levels was accepted as statistically significant. In the “cluster level” section at left, the corrected *p* values and the brain lobes with hypometabolism are reported. In the “peak level” section at right, the z-scores and peak coordinates, as well as the corresponding cortical regions and BAs, are reported

**Fig. 3:**
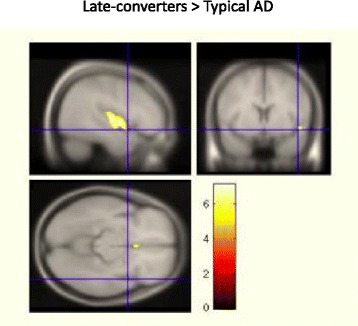
Brain metabolism in late converters compared with patients with “typical” Alzheimer’s disease (AD). When directly compared with “typical” AD, late-converter patients were characterized by two clusters of relatively preserved metabolism in the right middle temporal gyrus (Brodmann area [BA] 21) and in the left orbitofrontal cortex (BA 47). *See* Table 5 for details on coordinates and z-scores. Other details are the same as in Fig. 2 legend

Finally, when directly compared with “typical” AD, late converter patients were characterized by two clusters of relatively preserved metabolism in the right middle temporal gyrus (BA 21) and in the left orbitofrontal cortex (BA 47). *See* Fig. 3 and Table 5 for further details.

**Table 5:** Comparison between late-converter patients and patients with “typical” Alzheimer’s disease

	Cluster level		Peak level					
Cluster extent	Corrected *p* value	Cortical region	Maximum z-score		Talairach coordinates		Cortical region	BA
1483	0.005	R-temporal	6.53	67	− 34	− 12	Middle temporal gyrus	21
		L-frontal	5.51	− 46	9	18	Inferior frontal gyrus	47

*BA* Brodmann area

*p* < 0.05 corrected for multiple comparisons with the family-Wise error correction option at both peak and cluster levels was accepted as statistically significant. In the “cluster level” section at left, the corrected *p* value and the brain lobe with hypometabolism are reported. In the “peak level” section at right, the z-score and peak coordinates as well as the corresponding cortical region and BA are reported. Late-converter patients and those with “typical” AD are patients with mild cognitive impairment who clinically converted to dementia of Alzheimer‘s type at different times during follow-up (*see further details in the main text*)

#### Group B

Similarly, in the AMY+ MCI group, aggressive AMY+ MCI was characterized by a bilateral extended area of hypometabolism in the left superior temporal gyrus and the posterior cingulate cortex, as well as in the posterior parietal cortex, lateral cuneus, and precuneus in both hemispheres (BAs 7, 18, 19, 22, 31, and 40), whereas patients with smoldering MCI were characterized by hypometabolism limited to smaller clusters in the bilateral posterior parietal cortex (BA 40). In both cases, these regions substantially overlapped with the regions highlighted in the analyses performed on group A in patients with “typical” AD and in late-converter patients with MCI, respectively. Patients with AMY+ MCI belonging to the second tertile of group B showed a cluster of hypometabolism substantially overlapping the cluster highlighted in aggressive MCI (*see* Additional file 2: Figure S1). No significant differences were highlighted when we directly compared aggressive with smoldering AMY+ MCI. *See* Fig. 2 and Additional file 3: Table S1 for further details.

### Topography and extent of neurodegeneration in education-based subgroups

**Fig. 4:**
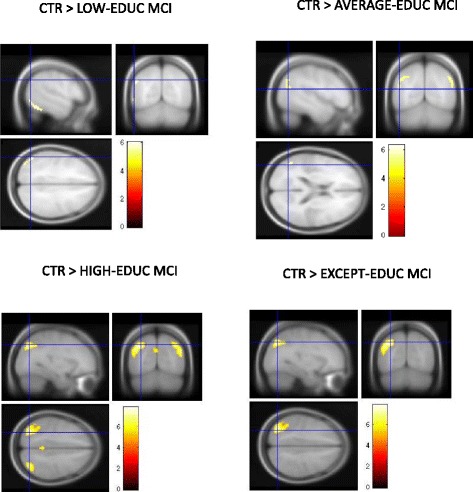
Cortical hypometabolism in education-based subgroups with respect to healthy volunteer control subjects (CTR). The whole patient group (*n* = 133) was divided into quartiles to obtain education-based subgroups: poorly educated (first quartile; Low-EDUC), average education level (second quartile; Average-EDUC), highly educated (High-EDUC; third quartile), and exceptionally educated (fourth quartile; Except-EDUC). All education-based subgroups were characterized by hypometabolism in Alzheimer’s disease-typical cortical regions with respect to CTR. However, the High-EDUC subgroup demonstrated a more extended hypometabolism (than the Low-Educ and Average-EDUC subgroups) involving a more extended hypometabolism in the left superior temporal and posterior cingulate cortices, in the right fusiform gyrus, and in the posterior parietal cortex and precuneus in both hemispheres. The Except-EDUC subgroup showed a cluster of significant hypometabolism including only the left posterior parietal cortex (larger with respect to Low- and Average-EDUC subgroups but not further extended with respect to the High-EDUC subgroup). *See* Table 6 for details on coordinates and z-scores. Other details are the same as in the Fig. 2 legend

When compared with CTR, all education-based subgroups were characterized by hypometabolism in AD-typical posterior parietal regions of both hemispheres. In particular, as expected, in the Low-EDUC and Average-EDUC subgroups, hypometabolism was restricted to the left posterior parietal and middle temporal cortices (BAs 40 and 38), whereas the High-EDUC subgroup demonstrated a more extended hypometabolism in the left superior temporal and posterior cingulate cortices, in the right fusiform gyrus, and in the posterior parietal cortex and precuneus in both hemispheres (BAs 7, 20, 22, 30, 31, and 40). When compared with CTR, the Except-EDUC subgroup showed a cluster of significant hypometabolism including the left posterior parietal cortex (larger with respect to Low- and Average-EDUC subgroups but not further extended with respect to High-EDUC subgroup). *See* Fig. 4 and Table 6 for further details.

**Table 6:** Whole-brain voxel-based analyses of ^18^F-fluorodeoxyglucose positron emission tomographic images in SPM8

	Cluster level		Peak level					
Cluster extent	Corrected *p* value	Cortical region	Maximum z-score		Talairach coordinates		Cortical region	BA
Comparison between CTR and poorly educated patients with MCI								
983	0.005	L-parietal	6.83	− 57	− 38	50	Inferior parietal lobule	40
		L-temporal	4.36	− 44	4	− 37	Middle temporal gyrus	38
Comparison between CTR and patients with MCI with an average education level								
300	0.008	L-parietal	6.2	− 54	− 33	49	Inferior parietal lobule	40
278	0.005	R-parietal	65.8	50	− 45	29	Inferior parietal lobule	40
Comparison between CTR and highly educated patients with MCI								
14,247	0.001	L-parietal	6.83	− 12	− 47	34	Precuneus	31
		R-parietal	5.00	10	− 64	47	Precuneus	7
		L-parietal	4.10	− 61	− 53	34	Supramarginal gyrus	40
		R-temporal	3.55	50	− 13	− 25	Fusiform gyrus	20
		L-temporal	4.08	− 65	− 44	10	Superior temporal gyrus	22
		L-parietal	4.01	− 53	− 37	51	Inferior parietal lobule	40
		L-limbic	3.73	− 18	− 58	16	Posterior cingulate gyrus	30
Comparison between CTR and exceptionally educated patients with MCI								
998	0.001	L-parietal	6.53	− 12	− 47	34	Precuneus	31
		L-parietal	5.08	− 61	− 53	34	Supramarginal gyrus	40

*Abbreviations: BA* Brodmann area, *CTR* Healthy volunteer control subjects, *MCI* Mild cognitive impairment

*p* < 0.05 corrected for multiple comparisons with family-wise error correction option at both peak and cluster levels was accepted as statistically significant. In the “cluster level” section at left, the corrected *p* value and the brain lobe with hypometabolism are reported. In the “peak level” section at right, the z-score and peak coordinates, as well as the corresponding cortical region and BA, are reported

## Discussion

The present findings support the value of baseline brain metabolism as a progression biomarker in MCI-AD despite the effect of reserve-related mechanisms. In fact, on one hand, highly educated patients demonstrated a level of cognitive impairment similar to that of poorly educated patients despite a more extended hypometabolism in posterior AD-typical regions. On the other hand, late converter patients with MCI-AD and patients with smoldering AMY+ MCI demonstrated a less extended and severe hypometabolism compared with patients with typically progressing MCI-AD. These findings were actually not in contrast to one another, because we highlighted that not the posterior parietal (cognitive reserve-related) regions but specifically the right temporal cortex and in particular the middle temporal gyrus were relatively spared in both the late-converter MCI-AD and smoldering AMY+ MCI subgroups. Ewers and colleagues previously aimed to examine the effect of education on brain metabolism in subjects with preclinical AD, and in keeping with our results, they highlighted a significant interaction between education and CSF Aβ_42_ status in posterior cingulate cortex and angular gyrus ROIs but not in the inferior and middle temporal gyri [14]. It was also recently demonstrated that the annual changes in tau tracer binding in middle and inferior temporal gyri are significantly related to episodic memory impairments in AD [33, 34]. More interestingly, in keeping with our results, a recent combined tau-PET and FDG-PET study demonstrated that decreased FDG uptake (but not tau tracer increased uptake) in the middle and inferior temporal gyri significantly predicted decreased global functioning as assessed by MMSE score [35].

Altogether, present and previous findings support the existence of a relatively homogeneous AD progression-related pattern of hypometabolism despite the well-known AD heterogeneity mirrored by tau-PET [35, 36]. In this framework, whereas previous findings highlighted the relevance of the middle and inferior temporal gyri in biological progression of patients with AD, they did not specifically address the role of these cortical regions in late-converting patients and/or in patients with smoldering MCI [9, 20, 37]. In the present study, less extended and severe hypometabolism was demonstrated in these regions in patients with slowly progressing MCI compared with those with typically progressing MCI [9, 20, 37]. It was previously hypothesized that the early diagnostic relevance of the posterior parietal hypometabolic pattern would render it less sensitive to further biological progression of the disease by demonstrating a sort of floor effect. This floor effect would not be shared by those areas with less severe hypometabolism, such as the inferior and middle temporal cortices [10, 38]. In other words, regions included later in the AD metabolic signature might show a more linear relationship with the advancing severity of disease, thus representing more sensitive markers of disease progression [20, 39, 40]. Interestingly enough, the prognostic value of brain metabolism in the middle and inferior temporal cortices was previously demonstrated to be independent of the main demographic/clinical variables known to influence symptom onset and progression to AD dementia, such as age, education level, and baseline MMSE score [10]. However, the role of these cortical regions as potential regions of resilience versus regions specifically targeted by more aggressive AD phenotypes was not specifically addressed [41]. In this framework, whereas *reserve* generally refers to the capability of sustaining cognition against AD-related damage even at high levels of AD pathology [11], *resilience* corresponds to the supposed capability of some individuals to slow the progression of neurodegeneration despite harboring the primary risk factors for the disease (e.g., advanced age and carriage of one or more ε4 alleles of the apolipoprotein E [APOE] gene) [41]. The present study thus seems to support the role of middle and inferior temporal gyri as sites of resistance (when relatively preserved) rather than as a hallmark of a more aggressive pattern of neurodegeneration (when hypometabolic). In this view, whereas educational attainment that occurs during sensitive periods of brain development is considered to have a significant impact on AD biomarker trajectories, lifestyle has increasingly been recognized as protective against cognitive decline in the elderly [42–46]. However, although these factors are associated with better cognitive performance, there is no clear consensus regarding their influence on ongoing AD pathophysiology. In the present study, the cortical regions whose “metabolic resistance” was associated with slower clinical progression had different localization with respect to the regions affected by education-related reserve. Accordingly, the role (or the concurrent role) of other protective factors sustaining resistance against neurodegeneration in the middle and inferior temporal gyri should be specifically addressed in the future.

Finally, we acknowledge some limitations of this study. First, because APOE genotype was not available in all patients, we could not perform specific analyses based on APOE subgroups. Another limitation is that the MMSE score might not be the most accurate tool to represent cognitive decline in our groups of patients with MCI. However, other individual tests were not available in all patients, because the neuropsychological battery changed over time and was often tailored according to the clinical characteristics of patients. Accordingly, the diversity of administered cognitive tests represents a further potential drawback of the present analysis. However, whereas a composite z-score-based neuropsychological index, when available, might better express the cognitive decline with the course of time, in this study we tried to accurately measure the annual rate of MMSE speed of reduction by (1) including only MMSE scores obtained during a proper neuropsychological evaluation by the neuropsychologist, (2) taking into account at least three different MMSE evaluations over time, and (3) correcting the results for the effect of age and baseline MMSE score. It should also be underlined that although we have in vivo confirmation of brain amyloidosis in patients belonging to group B, we lack an amyloidosis biomarker in most patients belonging to group A. In this group, the confirmation of AD was based on the results of neuropsychological examination and MRI and ^18^F-FDG-PET examinations at baseline and then clinically confirmed at the time of clinical diagnosis of dementia of Alzheimer’s type and in the further clinical follow-up. Therefore, we believe the risk of misdiagnosis was minimized. Finally, we chose not to correct PET results for partial volume effect (PVE), and the highlighted findings might at least partially be due to the concomitant underlying atrophy. Whereas this lack of correction might have influenced (i.e., magnified) some of the results, especially in case of small clusters such as in the comparison between CTR and poorly educated patients with MCI, it does not change the overall interpretation of our findings. In fact, the possible underlying atrophy is even a further sign of the neurodegeneration process. Moreover, in previous studies, the PVE correction has not substantially changed the results or even magnified the metabolic deficit [47–49].

## Conclusions

The present study suggests that the effect of education on brain metabolism may act through both reserve and resilience mechanisms in different brain regions possibly affecting the speed of progression from MCI to AD dementia stage. In fact, not the posterior parietal (cognitive reserve-related) regions but specifically the middle and inferior temporal gyri seem to be relatively spared in patients with slowly progressing MCI-AD. These findings thus support the existence of a relatively homogeneous AD progression-related pattern of hypometabolism despite AD heterogeneity and interference of cognitive reserve. Further larger studies are needed to assess whether these regions represent a more specific and topographically restricted target to test the effect of lifestyle enrichment and lifestyle-related risk factors in patients with MCI-AD.

## Additional files

Additional file 1: Supplementary Methods and Results. (DOCX 19 kb)

Additional file 2: Clusters of MMSE reduction. (PNG 66 kb)

Additional file 3: Supplementary Table. (DOCX 22 kb)

## Abbreviations

AD: Alzheimer’s disease
AMY+ MCI: Patients with mild cognitive impairment who had a positive result for a brain amyloidosis biomarker
AMY-PET: Amyloid positron emission tomography
APOE: Apolipoprotein E
Aβ_42_: amyloid-β 42
BA: Brodmann area
CDR: Clinical Dementia Rating
CSF: Cerebrospinal fluid
CTR: Healthy volunteer control subjects
ELISA: Enzyme-linked immunosorbent assay
Except-EDUC: Exceptionally educated
FDG-PET: ^18^F-Fluorodeoxyglucose positron emission tomography
High-EDUC: Highly educated
Low-EDUC: Poorly educated
MCI: Mild cognitive impairment
MCI-AD: Patients with mild cognitive impairment due to Alzheimer’s disease
MMSE: Mini Mental State Examination
MNI: Montreal Neurological Institute
MRI: Magnetic resonance imaging
NPS: Neuropsychology
PVE: Partial volume effect
ROI: Region of interest
SPM: Statistical Parametric Mapping
SUVR: Standardized uptake value ratio
ΔMMSE: Mini Mental State Examination score reduction
