# Supplementary material for: Metabolic correlates of reserve and resilience in MCI due to Alzheimer's Disease (AD)
Source: Alzheimers Res Ther. 2018 Apr 3;10:35. doi: 10.1186/s13195-018-0366-y (PMC5883593; doi:10.1186/s13195-018-0366-y)
Supplement: Supplementary file 1 — Supplementary Methods and Results. (DOCX 19 kb) [file 13195_2018_366_MOESM1_ESM.docx]

Methods

FDG PET Acquisition protocol

Subjects fasted for at least six hours. Before radiopharmaceutical injection, blood glucose was checked and was <7.8 mmol/l in all cases. After 10 minutes rest in a silent and obscured room, with eyes closed and ears unplugged, subjects were injected with approximately 200 MBq of 18F-FDG via a venous cannula. They remained in the room for 30 minutes after the injection and then moved to the PET room where scanning started approximately 45 minutes after the injection. A polycarbonate head holder was used to reduce head movements during the scan. Images were acquired by means of a SIEMENS Biograph 16 PET/CT equipment with a total axial field of view of 15 cm and no interplane gap space. Scan acquisition time was 15 minutes with 3-dimensional mode. Images were reconstructed through an ordered subset-expectation maximization algorithm, 16 subset and 6 iterations, with a reconstructed voxel size of 1.33×1 .33×2.00 mm. Attenuation correction was based on CT scan. Dicom files were exported and converted into Analyse files.

Amyloid PET protocol

Doses of AMY-PET tracers and tracer uptake time after injected followed manufacturer instructions. Therefore 370, 300 and 185 MBq was injected for AMY-PET imaging 18F-Florbetapir, 18F-Florbetaben and 18F-Flutemetamol respectively. Images were acquired 50 minutes post injection for 18F-Florbetapir and 90 minutes for 18F-Florbetaben and 18F-Flutemetamol . Attenuation was corrected by CT scan. Then the images were reoriented to anterior commissure-posterior commissure with the same matrix size and voxel size so that all camera models presented images of similar orientation and appearance in the viewer, and were then passed on to the image visual interpretation according to manufacturer’s instructions.

SUV ratio computation

We calculated the average cortico-cerebellar SUVr on AMY-PET scans of patients belonging to group B as described in references 28 of the main manuscript. Briefly, we used the whole cerebellum (white and gray matter) as reference and the average of a number of cortical regions of interest (ROIs) as uptake regions. The cortical regions were: medial frontal gyrus, lateral frontal cortex (middle frontal gyrus), lateral temporal cortex (middle temporal gyrus), lateral parietal cortex (inferior parietal lobule), insula, caudate nucleus, and precuneus-posterior cingulate region. Scan positivity was confirmed using published SUVr cut-offs for each tracer (see references 26 and 27 of the main manuscript). SUVr was higher than 1.4 in all patients.

Cerebrospinal Fluid Assay

The CSF samples were collected in polypropylene tubes. The CSF samples were directly transported to the laboratory, centrifuged, and measured or stored at –80◦C until use.

From one frozen aliquot of CSF, the assays were run in parallel according to the manufacturer’s instructions. Each sample was assessed in duplicate. A sigmoidal standard curve was plotted to allow the quantita- tive expression (pg mL_1) of measured light absorbance.

Below in Table B we reported the results of the CSF assay in the 9 patients whose amyloid status was assessed by means of CSF assay.
