# Supplementary figures and images for: Metabolic correlates of reserve and resilience in MCI due to Alzheimer's Disease (AD)
Source: Alzheimers Res Ther. 2018 Apr 3;10:35. doi: 10.1186/s13195-018-0366-y (PMC5883593; doi:10.1186/s13195-018-0366-y)

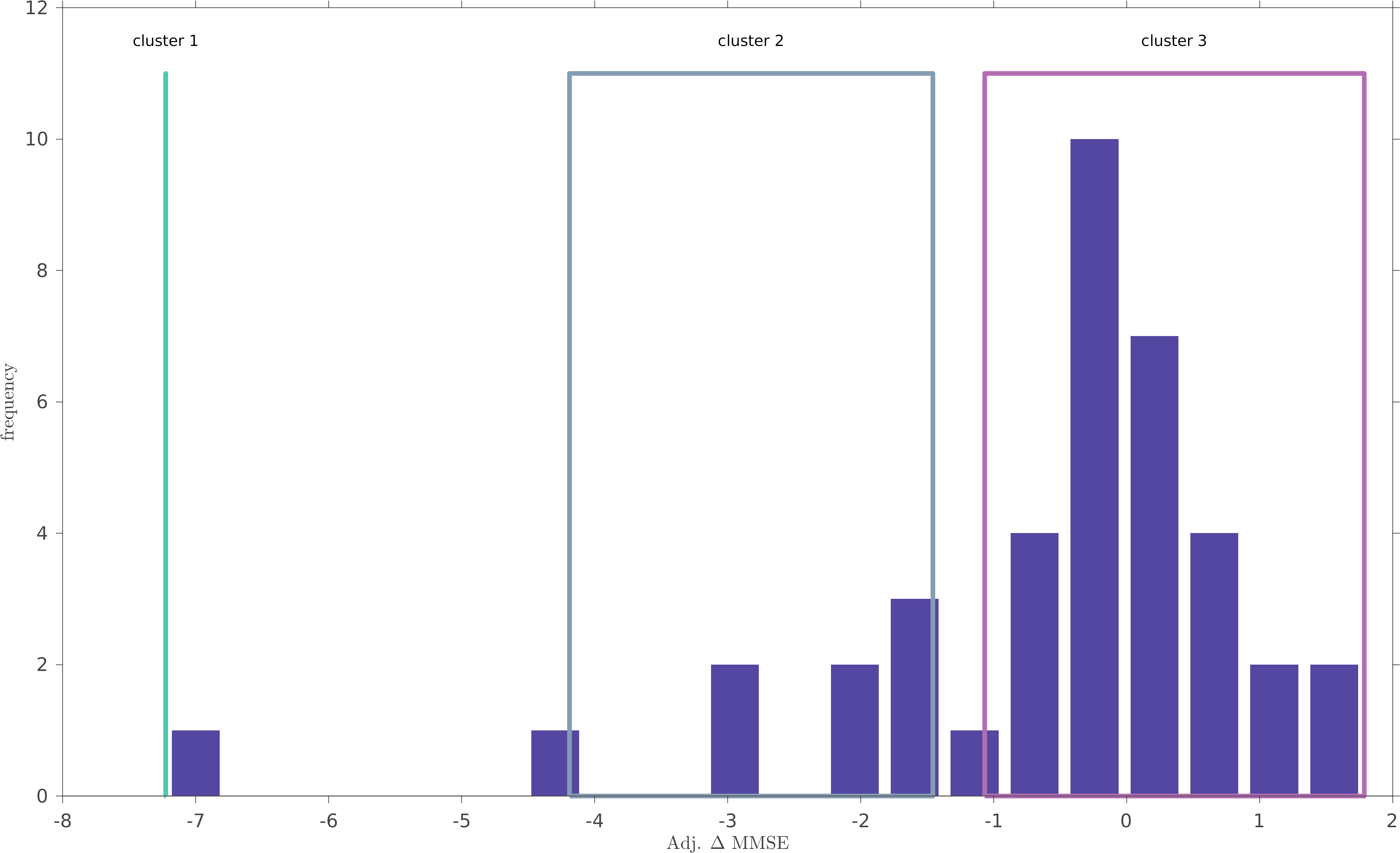

Supplement: Supplementary file 2 — Clusters of MMSE reduction. (PNG 66 kb) [file 13195_2018_366_MOESM2_ESM.png]
