# Supplementary material for: Metabolic correlates of reserve and resilience in MCI due to Alzheimer's Disease (AD)
Source: Alzheimers Res Ther. 2018 Apr 3;10:35. doi: 10.1186/s13195-018-0366-y (PMC5883593; doi:10.1186/s13195-018-0366-y)
Supplement: Supplementary file 3 — Supplementary Table. (DOCX 22 kb) [file 13195_2018_366_MOESM3_ESM.docx]

**Table S1: Whole brain voxel-based analyses of ^18^F-FDG PET images in SPM8.**

1. **Comparison between Controls and aggressive AMY+ MCI.**

|  | **Cluster Level** |  | **Peak Level** |  |  |  |  |  |
| --- | --- | --- | --- | --- | --- | --- | --- | --- |
| Cluster Extent | Corrected P value | Cortical region | Maximum Z score |  | Talairach Coordinates |  | Cortical Region | BA |
| 10232 | 0.0001 | L-Limbic | 5.01 | -10 | -43 | 39 | Posterior Cingulate Gyrus | 31 |
|  |  | L-Parietal | 5.04 | -42 | -74 | 44 | Precuneus | 19 |
|  |  | L-Parietal | 4.98 | -44 | -68 | 49 | Inferior Parietal Lobule | 7 |
|  |  | R-Parietal | 4.89 | 50 | -47 | 26 | Inferior Parietal Lobule | 40 |
|  |  | L-Parietal | 4.73 | -12 | -47 | 34 | Precuneus | 31 |
|  |  | R-Occipital | 4.24 | 30 | -75 | 24 | Precuneus | 31 |
|  |  | R-Parietal | 4.19 | 10 | -64 | 47 | Precuneus | 7 |
|  |  | L-Parietal | 4.08 | -61 | -53 | 34 | Supramarginal Gyrus | 40 |
|  |  | L-Temporal | 4.08 | -65 | -44 | 10 | Superior Temporal Gyrus | 22 |
|  |  | R-Occipital | 3.88 | 8 | -94 | 27 | Cuneus | 19 |
|  |  | L-Occipital | 3.85 | -26 | -92 | 30 | Cuneus | 19 |
|  |  | L-Parietal | 3.81 | -57 | -38 | 50 | Inferior Parietal Lobule | 40 |
|  |  | L-Occipital | 3.73 | -30 | -92 | 25 | Superior Occipital Gyrus | 19 |

1. **Comparison between Controls and smoldering AMY+ MCI.**

|  | **Cluster Level** |  | **Peak Level** |  |  |  |  |  |
| --- | --- | --- | --- | --- | --- | --- | --- | --- |
| Cluster Extent | Corrected P value | Cortical region | Maximum Z score |  | Talairach Coordinates |  | Cortical Region | BA |
| 1283  967 | 0.008  0.003 | R-Parietal | 5.89 | 50 | -47 | 30 | Inferior Parietal Lobule | 40 |
|  |  |  |  |  |  |  |  |  |
|  |  | L-Parietal | 3.83 | -57 | -38 | 50 | Inferior Parietal Lobule | 40 |

p <0.05, corrected for multiple comparisons with the Family-Wise-error option at both peak and cluster level were accepted as statistically significant. In the ‘cluster level’ section on the left, the corrected p value and the brain lobe with hypometabolism are reported. In the ‘peak level’ section on the right, the Z score and peak coordinates, the corresponding cortical region and Brodmann area (BA) are reported. AMY+ MCI, mild cognitive impairment patients with brain amyloidosis; L, left; R, right.
